# Welcome to the TRIBE: An approach to identify RNA ligands of RNA-binding proteins in rice

**DOI:** 10.1093/plphys/kiad175

**Published:** 2023-03-21

**Authors:** Manuel González-Fuente

**Affiliations:** Plant Physiology, American Society of Plant Biologists, USA; Faculty of Biology & Biotechnology, Ruhr-University Bochum, Bochum D-44780, Germany

RNA plays a vital role as the central and necessary intermediary in the flow of genetic information between DNA and proteins. Throughout their lifecycle, RNA molecules are accompanied by a multitude of RNA-binding proteins (RBPs). RBPs mediate the transcription, processing, export, translation, and degradation of RNA molecules. To perform all these functions, living organisms possess a large number of RBPs, with more than 800 in plant species (Marondedze 2020).

**Figure 1.**
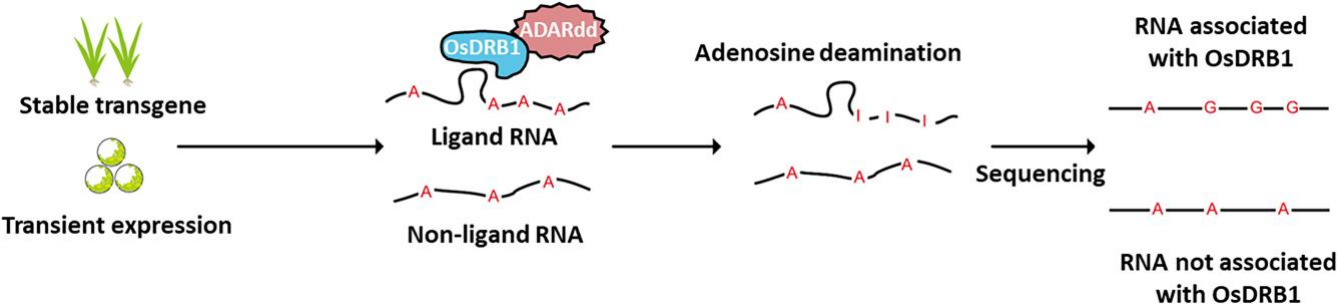
TRIBE approach in rice.

To understand how a given RBP modulates the function and fate of RNA molecules, it is crucial to identify which RNA molecules the RBP binds. Traditionally, RBP–RNA interactions have been discovered using indirect approaches or in vitro. These low-throughput techniques were later complemented with higher-throughput and in vivo alternatives such as RNA immunoprecipitation sequencing (RIP-seq) and subsequent improvements (Burjoski and Reddy 2021). These approaches rely on the availability of suitable antibodies and the efficiency of crosslinking, which limits their application in plants (Zhou et al. 2021). An alternative method called TRIBE, which stands for *t*argets of *R*BPs *i*dentified *b*y *e*diting, was developed in the animal field (McMahon et al. 2016) and recently applied to Arabidopsis (*Arabidopsis thaliana*) (Arribas-Hernández et al. 2021; Zhou et al. 2021). This method uses protein fusions of candidate RBPs with the deaminase domain of adenosine deaminase acting on RNA (ADARdd). ADARdd specifically deaminates adenosines from RNA molecules bound to the RBP, transforming them into inosines (A-to-I), which can be later recognized by sequencing as A-to-G and T-to-C changes when comparing the transcriptome to the reference genome (Figure 1).

In this issue of *Plant Physiology*, Yin et al. (2023) defined the RNA editing properties of ADARdd in plants and developed a stringent pipeline to confidently identify ADARdd-mediated editing sites from RNA sequencing (RNA-seq) data. As a proof of concept, they applied this strategy to rice (*Oryza sativa*) DOUBLE-STRANDED RNA BINDING PROTEIN 1 (OsDRB1) to identify putative RNA ligands, opening the door to use this technique in other plant species outside the model plant Arabidopsis.

To characterize the RNA editing properties of ADARdd in plants, the authors made use of 2 well-established RBP–RNA ligand pairs from a virus and Arabidopsis (Meyer et al. 2017; Yang et al. 2019). By coexpressing the viral RBP–ADARdd fusion with its known RNA ligand in rice protoplasts, the authors narrowed down the RNA editing window of ADARdd to 41 nucleotides from the RNA binding site. However, as the viral RNA tested was double stranded, the authors used the Arabidopsis RBP–RNA ligand pair to confirm that ADARdd also edited single-stranded transcripts (Meyer et al. 2017).

Once ADARdd was confirmed as functional in rice protoplasts and that it could target single-stranded RNA, the authors generated stable transgenic rice lines expressing ADARdd fused to OsDRB1 and conducted RNA-seq experiments with roots and leaves from adult plants. High-confidence editing sites were identified applying a stringent pipeline to the RNA-seq data. Interestingly, 25% to 50% of the identified editing sites were organ specific, suggesting important tissue specificity of OsDRB1–RNA interactions. These editing sites were located in almost 800 annotated rice transcripts. Most of these transcripts arose from repetitive elements and coding genes. In the case of coding genes, the editing sites were located predominantly in the noncoding parts (i.e. introns and terminal untranslated regions). As most small RNAs (sRNAs) are generated from noncoding regions of transcripts and from repetitive elements (Reich and Bass 2019), the authors performed sRNA sequencing on the transgenic rice plants to test whether OsDRB1–ADARdd could edit sRNAs as well. Indeed, high-confidence editing sites were observed in mature sRNAs, further supporting the involvement of OsDRB1 in sRNA genesis in rice (Raghuram et al. 2015) and proving that ADARdd-mediated RNA edits are maintained throughout sRNA processing.

In summary, Yin et al. (2023) described the RNA-editing properties of ADARdd in rice and developed a comprehensive and stringent pipeline to identify RNA ligands of an RBP by TRIBE/RNA-seq, using OsDRB1 as a proof of concept. This work supports previous notions suggesting that TRIBE is more sensitive than RIP-seq–derived approaches (McMahon et al. 2016; Arribas-Hernández et al. 2021). In addition to increased sensitivity, TRIBE is also less laborious and produces less background noise than alternative RIP-seq–derived approaches. However, the success of TRIBE depends on the use of adequate negative controls, sufficient replicates, and deep sequencing. Moreover, it should be demonstrated that the fusion protein maintains the original subcellular localization and that its expression is not detrimental to the plant. Together with previous pioneering works in Arabidopsis (Arribas-Hernández et al. 2021; Zhou et al. 2021), this study opens the door to applying this technique to any other genetically amenable plant species, overcoming some of the limitations of traditional standard approaches. Furthermore, the identified RNA ligands of OsDRB1 will help us better understand the molecular functions of this protein in rice and characterize its role in development and stress responses.

A schematic representation of the TRIBE approach was employed in this study to identify the RNA ligands of rice (*O. sativa*) OsDRB1. A fusion protein of OsDRB1 with the deaminase domain of ADARdd is expressed either stably in transgenic plants or transiently in protoplasts. ADARdd deaminates the adenosines of the RNA molecules bound to OsDRB1, transforming them into inosines that can be detected by sequencing, adapted from Yin et al. (2023).
